# Toward a wearable monitor of local muscle fatigue during electrical muscle stimulation using tissue Doppler imaging

**DOI:** 10.1017/wtc.2022.10

**Published:** 2022-07-20

**Authors:** Joseph A. Majdi, Samuel A. Acuña, Parag V. Chitnis, Siddhartha Sikdar

**Affiliations:** 1 Department of Bioengineering, George Mason University, Fairfax, Virginia, USA; 2 Center for Adaptive Systems of Brain–Body Interactions, George Mason University, Fairfax, Virginia, USA

**Keywords:** exoskeletons, feedback devices, human–robot interaction, performance characterization

## Abstract

Electrical muscle stimulation (EMS) is widely used in rehabilitation and athletic training to generate involuntary muscle contractions. However, EMS leads to rapid muscle fatigue, limiting the force a muscle can produce during prolonged use. Currently available methods to monitor localized muscle fatigue and recovery are generally not compatible with EMS. The purpose of this study was to examine whether Doppler ultrasound imaging can assess changes in stimulated muscle twitches that are related to muscle fatigue from electrical stimulation. We stimulated five isometric muscle twitches in the medial and lateral gastrocnemius of 13 healthy subjects before and after a fatiguing EMS protocol. Tissue Doppler imaging of the medial gastrocnemius recorded muscle tissue velocities during each twitch. Features of the average muscle tissue velocity waveforms changed immediately after the fatiguing stimulation protocol (peak velocity: -38%, p = .022; *time-to-zero velocity*: +8%, p = .050). As the fatigued muscle recovered, the features of the average tissue velocity waveforms showed a return towards their baseline values similar to that of the normalized ankle torque. We also found that features of the average tissue velocity waveform could significantly predict the ankle twitch torque for each participant (R^2^ = 0.255–0.849, p < .001). Our results provide evidence that Doppler ultrasound imaging can detect changes in muscle tissue during isometric muscle twitch that are related to muscle fatigue, fatigue recovery, and the generated joint torque. Tissue Doppler imaging may be a feasible method to monitor localized muscle fatigue during EMS in a wearable device.

## Introduction

Electrical muscle stimulation (EMS) is an increasingly popular approach to train, rehabilitate, and evaluate muscle contraction using electrical impulses. EMS (which encompasses both functional electrical stimulation and neuromuscular electrical stimulation) is widely used in a variety of applications, such as a strength training tool to improve athletic performance (Lake, 1992; Babault et al., 2011), as a rehabilitative tool to improve motor function for individuals with motor deficits (Rossato et al., 2009; Angeli et al., 2014; Cuesta-Gómez et al., 2017; D’Anna et al., 2017; Laubacher et al., 2019; Spieker et al., 2020), and as a research tool to investigate muscular function in vivo (Maffiuletti et al., 2011; Doucet et al., 2012). A typical EMS system generates patterns of electrical impulses and delivers them to muscle fibers via electrodes, which are commonly adhered to skin over the targeted muscle motor points. The target muscles involuntarily contract in response to the stimulation pattern, thus providing a user extensive control over the timing and amplitude of the generated induced muscle forces. However, most EMS systems use open-loop control of their stimulation patterns and fail to account for real-time changes in muscle performance, such as muscle fatigue.

Muscle fatigue, the decreased capacity of a muscle to generate force after exercise (Wan et al., 2017), is a primary consideration for EMS because stimulated muscles tend to fatigue very rapidly (Ibitoye et al., 2016; Buckmire et al., 2018; Schmoll et al., 2021). During voluntary muscle contraction, motor units are activated asynchronously (Bickel et al., 2011) with a discharge rate proportional to the desired level of contraction (Del Vecchio et al., 2019). However, EMS elicits unnatural activation and discharge of the motor units, causing stimulated muscles to fatigue faster (Binder-Macleod and Snyder-Mackler, 1993). This remains a major limitation for many applications of EMS, such as during strength training (Gondin et al., 2011) or physical rehabilitation (Ibitoye et al., 2016). Further, without adequate rest to recover from fatigue, overworked muscles are at risk for injury and lead to muscle soreness and pain (Dugan and Frontera, 2000). Attempts to mitigate EMS-induced muscle fatigue often involve spatially distributing the stimulation (Sayenko et al., 2014; Buckmire et al., 2018; Ye et al., 2021) and modifying the stimulation parameters (e.g., timing and waveforms) (Graham et al., 2006; Shimada et al., 2006). However, these open-loop approaches do not attempt to evaluate or characterize the induced muscle fatigue throughout the stimulation protocol. EMS protocols would greatly benefit from methods to monitor muscle fatigue and recovery within individual muscles.

Due to the ease of implementation and abundance of signal features, surface electromyography (sEMG) has emerged as the primary method to assess localized muscle activity and fatigue (Edwards and Lippold, 1956; Shi et al., 2007; Cifrek et al., 2009; Al-Mulla et al., 2011; Yousif et al., 2019). However, sEMG is poorly suited for monitoring muscle fatigue during EMS because, in addition to interference and crosstalk from adjacent muscles, sEMG is extremely sensitive to electrical interference (Tankisi et al., 2020). sEMG records electrical activity during muscle contraction via electrodes placed on the skin, which are by necessity near the electrodes used in EMS. EMS impulses can severely corrupt the sEMG signal with artifacts (Mandrile et al., 2003). For example, EMS can deliver impulses on the order of 100 V while sEMG attempts to record muscle signals that are on the order of <100 mV with an inherently low SNR. Although advanced signal processing methods to remove stimulation artifacts from sEMG signals are emerging (Zhang et al., 2011; Liu et al., 2014; Jung et al., 2021), working with sEMG signals during EMS has remained challenging. Thus, muscle fatigue monitoring during EMS would greatly benefit from a robust alternative to sEMG.

Various noninvasive techniques have attempted to quantify localized muscle fatigue using methods that are not as susceptible to electrical interference (Al-Mulla et al., 2011), such as mechanomyography (Uwamahoro et al., 2021), near-infrared spectroscopy (Yoshitake et al., 2001), and sonomyography (Zhang et al., 2022b). Mechanomyography can be considered the mechanical equivalent of sEMG by recording low-frequency (i.e., 2–200 Hz) surface vibrations produced by muscle fibers during contraction (Islam et al., 2013), but it has limited utility due to its sensitivity to acoustic/vibrational interference during movement (Woodward et al., 2019). Near-infrared spectroscopy can provide a measure of oxygenation in a fatiguing muscle but is also very sensitive to movement and is therefore mainly used as additional information to complement sEMG (Al-Mulla et al., 2011; Scano et al., 2020). Sonomyography uses ultrasound imaging to describe localized morphological changes in muscle and remains an active area of research (Sikdar et al., 2009; Al-Mulla et al., 2011; Sikdar et al., 2014; Akhlaghi et al., 2016). Ultrasound imaging is a common, noninvasive clinical tool compatible with EMS and has shown utility for quantifying muscle architecture (Shi et al., 2012; Franz et al., 2015; Dieterich et al., 2017), mechanical activation (Ito et al., 1998; Castellini et al., 2012; Shi et al., 2012; Sikdar et al., 2014; Akhlaghi et al., 2016), force (Hatta et al., 1988; Bakke et al., 1992; Koyama et al., 2005; Tsutsui et al., 2007), and fatigue (Witte et al., 2004, 2006; Shi et al., 2007; Sheng et al., 2019). Ultrasound imaging can also distinguish between deep and superficial muscles, whereas sEMG signals contain the summation of any electrical muscle activity beneath the electrode. Previous technical limitations (e.g., large form factor of hand-held ultrasound probes with tethered cables, need for custom holders to stabilize probes during ambulatory tasks, and real-time data processing and transmission) have inhibited its use in freely moving subjects, but recent developments have shown remarkable potential for wearable devices capable of sonomyography (Sikdar et al., 2014; Majdi et al., 2018; AlMohimeed and Ono, 2020). Thus, monitoring real-time changes in localized muscle fatigue during EMS might be possible using sonomyographic data.

Although sonomyography has typically been used to track muscle motion using B-mode (2 dimensional image sequences) and M-mode images (one scan line over time), tissue Doppler imaging (TDI) is another ultrasound mode for quantifying tissue motion (Sikdar et al., 2014). This approach extracts tissue velocities and associated metrics such as strain rate by measuring the phase shift of the ultrasonic reflections in rapid (kHz) succession. Unlike computationally intensive postprocessing motion analyses like speckle tracking using B-mode images, RF data, analytical signals, and so forth (Witte et al., 2004; Franz et al., 2015), TDI can quantify local tissue velocities in real time with sub-mm detail with conventional Doppler processing methods. TDI has proven to be very sensitive to the motion of myocardial structures, and thus this approach is commonly used clinically to assess myocardial dysfunction (Weidemann and Strotmann, 2008). However, only a few studies have explored using TDI to assess the dynamic functions of skeletal muscle (Campbell et al., 2005; Koh and McNally, 2007). For example, TDI can reliably detect the mechanical onset of muscle activity (Pulkovski et al., 2008). TDI can also distinguish muscle contraction from passive muscle motion (Grubb et al., 1995). However, the use of TDI to quantify changes to skeletal muscle experiencing fatigue due to EMS remains unexplored.

Our ultimate goal is to develop a wearable, real-time monitor of EMS-induced muscle fatigue to characterize time-varying EMS/muscle performance. Our overarching hypothesis is that TDI is sensitive to the mechanical signs of muscle fatigue during EMS and has the potential to monitor muscle fatigue and recovery in real time. As a first step, the purpose of this study was to examine whether TDI can assess changes in stimulated muscle twitches that are related to muscle fatigue from electrical stimulation. We used TDI to extract gastrocnemius muscle tissue velocities during isometric muscle twitches before and after a fatiguing EMS protocol. We first hypothesized that muscle tissue velocities during stimulated muscle twitches would decrease after the fatiguing EMS protocol, and then gradually increase during fatigue recovery. Our second hypothesis was that changes to the muscle tissue velocities would be related to increases in isometric muscle torque during fatigue recovery.

## Methods

### Participants

We recruited healthy adults to participate in the study. The self-reported inclusion criteria were: (a) 18–60 years old, (b) able to consent and participate in this study, (c) able to walk independently, and (d) did not use a pacemaker or any other implanted electronic device. We excluded participants with any previous history of stroke, cerebral palsy, multiple sclerosis, or any other neuromuscular disorder. We also excluded participants with a history of cardiovascular disease, lower extremity surgery or injury, diagnosis of an unstable spine, unhealed limb of pelvic fractures, recurrent fractures, or osteoporosis. Any participants who currently had active pressure sores, open wounds, or an infection were also excluded. The experimental protocol was approved by the George Mason University Institutional Review Board, and each research subject provided written informed consent before participating in the study.

### Experimental Setup

We performed our assessments of muscle fatigue on subjects instrumented with a Biodex II dynamometer (Biodex Medical Systems, Upton, NY) retrofitted with a Humac interface (CSMi Solutions, Stoughton, MA). Subjects were placed in either a prone or seated position with either their right or left leg fitting into the Biodex, chosen at random by coin flip. The Biodex was configured such that subjects could perform isometric plantar flexion with the chosen leg. To prevent lateral ankle movement, the ankle was secured at 90° using a foot plate. For subjects in the prone position, we recorded the ankle torque from the Biodex using LabView (National Instruments, Austin, TX). For subjects in the seated position, we recorded ankle torque from a load cell (Futek Inc., Irvine, CA) affixed to the platform under the applied center of pressure.

**Figure 1.**
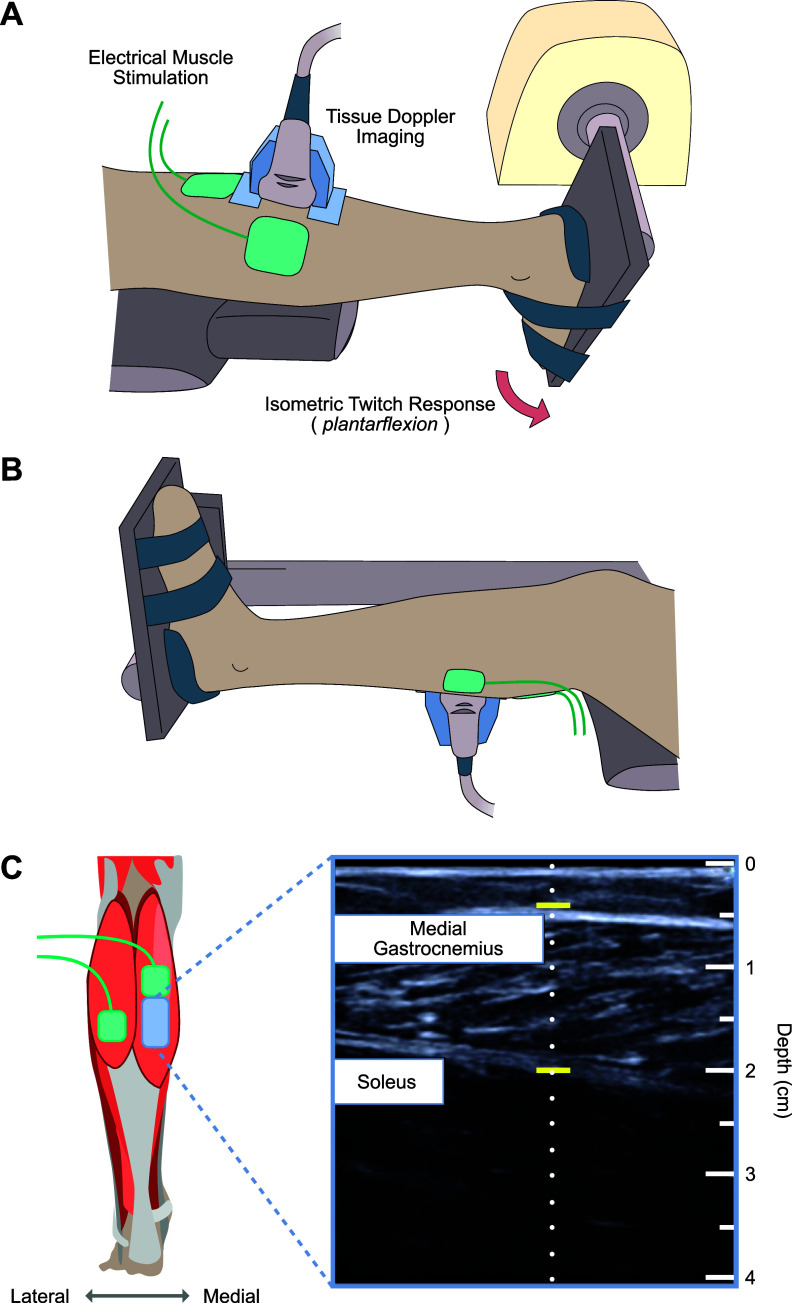
We used tissue Doppler imaging to examine gastrocnemius muscle tissue velocities during stimulated isometric muscle twitches before and after electrical muscle stimulation. Our experimental setup for (A) participants in a prone position and for (B) participants in a seated position. (C) An example longitudinal view B-mode ultrasound image of the medial gastrocnemius used during placement and orientation of the ultrasound probe. We adjusted the probe to create an optimal image of gastrocnemius muscle fibers. The thin dotted line in the middle of the B-mode image represents the scan line (where the beam is directed during the rapid TDI pulsing). The two horizontal yellow lines define the gating depth (i.e., the zone measured) and were set to cover the gastrocnemius muscle tissue. The major tick marks to the right of the image represent 1 cm, with minor tick marks at the 0.5 cm midpoints. The total image is just over 4 cm deep and 3.8 cm wide.

Subjects were instrumented with a 38 mm wide L 14–5 MHz ultrasound probe with a center frequency of 6.66 Mhz, attached to a SonixOne ultrasound system (BK Analogic, Richmond, BC) with a sample rate of 40 MHz, beamformed across 32 channels. The probe was placed as close as possible to the center of the muscle belly from the distal side of the medial gastrocnemius muscle belly (Figure 1). The probe was oriented over the muscle to allow for an optimal image of medial gastrocnemius fiber orientation. A custom 3D-printed mount held the probe perpendicular to the muscle, using a neoprene cuff secured around the calf. Ultrasound data was collected in the pulse-wave TDI mode. The upper and lower boundaries of the Doppler gate, which define the depths of interest along the TDI scan line (see Figure 1c), were set to cover the entire thickness of the medial gastrocnemius along the scan line at both rest and during voluntary flexion. We set the pulse repetition frequency to 10 kHz, but if the gate depth was small enough to permit the buffer to use a higher pulse repetition frequency, we increased the frequency to 12.5 kHz. Due to memory limits on the ultrasound machine, we chose not to image regions of the soleus. We also did not account for soleus activation or movement because the gastrocnemius has distinct innervation zones from the soleus and thus could be activated independently during stimulation (Nordez et al., 2009).

We placed two electrodes for an EMS system (Rehastim2, Hasomed GmbH, Magdeburg, GE) over the heads of the gastrocnemius in accordance with the manufacturer’s guidelines (Figure 1). The first electrode was placed over the medial gastrocnemius proximal to the ultrasound probe (at approximately the middle of the muscle belly). The second electrode was placed over the distal end of the lateral gastrocnemius. We stimulated both heads of the gastrocnemius to minimize unnatural lateral shifting of the muscle belly. We controlled the EMS system using a custom Matlab/Simulink interface (MathWorks Inc., Natick, MA).

### Experimental Protocol

We established a normalized stimulation intensity (I_20_) for each subject. First, we instructed subjects to generate a maximum voluntary isometric contraction (MVIC) during plantar flexion. We then electrically stimulated the gastrocnemius with pulse trains of increasing current (starting from 5 mA) until we determined the current necessary to stimulate the gastrocnemius to produce an isometric ankle torque at 20% of the MVIC torque (I_20_). All EMS used biphasic, constant current pulses with 100 μs pulse width per phase and 100 μs delay between phases. Fatiguing stimulation used 50 Hz pulse trains (20 ms main pulse interval) whereas muscle twitch stimulation used individual biphasic pulses.

**Figure 2.**
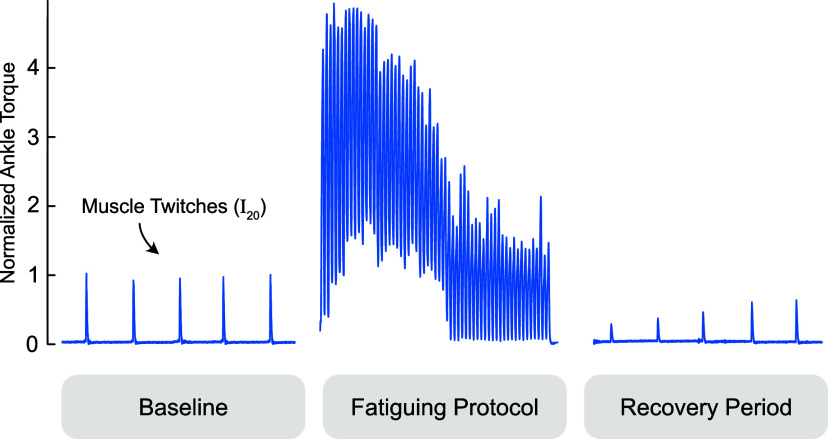
We examined tissue Doppler images before and after a fatiguing stimulation protocol pictured. The gastrocnemius is intermittently twitched to establish a baseline, intentionally fatigued with a one minute electrical muscle stimulation protocol, and intermittently twitched during a period of at least two minutes to evaluate fatigue recovery. Ankle torque is normalized to the average of the pre-fatigue twitch values and times are scaled for easier viewing.

We then recorded ankle torque and TDI muscle velocity during electrically stimulated muscle twitches before and after a fatiguing EMS protocol (Figure 2). We first established a baseline (i.e., prefatigue) muscle twitch behavior by stimulating the gastrocnemius at least five times with single pulses at the stimulation intensity I_20_. Subjects then received a time-varying stimulation train intended to emulate cyclic stepping and induce muscle fatigue, lasting a total of 60 s per fatiguing period. The stimulation train included 60 cycles (i.e., 60 steps). For each stimulation cycle, current was ramped up from zero to I_20_ over 0.12 s, held at I_20_ for 0.5 s, and then ramped down to zero for 0.12 s, followed by 0.26 s rest, for a total period of 1 s. After the fatiguing stimulation train, we assessed muscle twitch as the muscle recovered by electrically stimulating the medial gastrocnemius with single pulses at stimulation intensity I_20_ approximately once every 20 s over 2 min of rest. Due to memory limits of the ultrasound machine, we could only record for approximately 3–5 s at a time depending on the size of the Doppler gate (which depended on the thickness of the muscle measured) and pulse repetition frequency, plus approximately 10 s to save.

After we started collecting data using the fatiguing EMS protocol, we decided to examine how the TDI muscle velocities might change after multiple rounds of the fatiguing EMS protocol. For the subjects recruited after this decision was made, we included four additional rounds of the fatiguing EMS protocol separated by 2-min periods for rest.

### Data Analysis

For each TDI imaging period, we generated the average TDI velocity waveform over the thickness of the medial gastrocnemius tissue (i.e., across the Doppler gate) using an autocorrelation method (Shiina et al., 1996). Briefly, TDI is collected as a beamformed scanline acquired in rapid succession (kHz range). Scanlines are converted to their complex in-phase and quadrature components via a Hilbert transform, and an estimate of velocity is determined by comparing the relative change in phase angle between pulses for every sample along the imaging depth defined by the Doppler gate. Using the change in phase angle vector ( 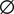 ), the velocity (*v*) for a given time (*t*), pixel depth (*d*), and period between pulses (*T*) can be calculated with the following equation:

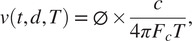

where *c* is the speed of sound and *F_c_* is the center frequency of the transducer. The resulting velocity field image is similar to an M-mode image, except each data point reflects the estimated velocity at a given depth and time point, rather than echo intensity. The data were downsampled to 1-kHz to emphasize low tissue velocities, and then averaged across depth to create a 1D velocity waveform for further analysis.

**Figure 3.**
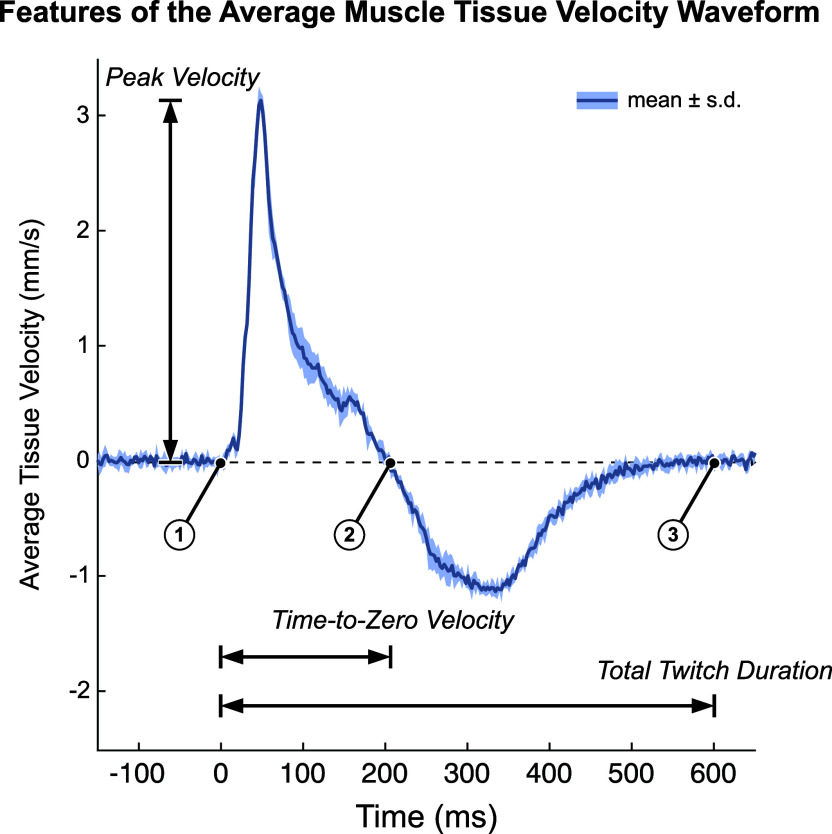
We quantified tissue Doppler imaging during a stimulated muscle twitch by extracting key features of the average muscle tissue velocity waveforms. Timepoint 1 is the onset of muscle twitch, time point 2 is the zero-crossing of tissue velocity waveform, and time point 3 is the end of muscle twitch. Several waveform features were used in this analysis. Time-To-Zero velocity is defined as time point 2 minus time point 1. We interpret this as the period of net expansion along the cross section of the muscle during a muscle twitch. Total twitch duration is defined as time point 3 minus time point 2. Peak Velocity is the maximum absolute velocity during the initial expansion phase (from time point1 to time point 2). The Peak Velocity-Time Integral (Peak VTI) is the integral of the average tissue velocity waveform, whose peak occurs at time point 2 (Peak VTI is not shown on the figure). Data plotted as the mean ± standard deviation of the recorded tissue velocities of a representative twitch response (pre-fatigue).

Average velocity waveforms were manually divided into three-time points: (a) the onset of muscle twitch, (b) the zero-crossing of tissue velocity (i.e., from positive to negative velocity or vice versa), and (c) the end of muscle twitch (i.e., when the velocity returns to zero). For the end point (point 3), spectral entropy (Misra et al., 2004) with a 164 sample window and cutoff of 0.7 was used to help guide the manual segmentation. During the first time interval, the velocity profile represents a net velocity measured through the muscle thickness which we interpret as the time during which the muscle undergoes net cross-sectional expansion. Conversely, we interpret the second time interval as the time during which the net expansion stops and the musculotendon unit passively contracts to baseline which necessarily is of opposite sign as the expansion period. Several features were then extracted from the average tissue velocity waveforms (Figure 3). The *peak velocity* was calculated as the maximum absolute velocity during the net (active) cross-sectional expansion (i.e., between the first and second time points). The *time-to-zero velocity* was calculated as the difference between the first and second time points. The *total twitch duration* was calculated as the difference between the first and third time points. The *peak velocity-time integral (VTI)* was calculated as the maximum value of the integral of the average velocity waveform, which occurs at the second time point, and represents the accumulation of net cross-section velocity as the muscle expands and releases. Unlike *peak velocity*, which depends on the fibers firing in synchrony, we believe the *peak VTI* to record the cumulative effects of fibers firing and more robust to any variations in electromechanical delay in the fibers. We also approximated the *peak tissue strain rate* by first smoothing the pixel-by-pixel velocities using a 1.925 mm (100-sample) moving average and then calculating the average spatial gradient over the depth of the gating window (which varied by subject). In nonisometric conditions, the muscle body could possibly shift toward or away from the probe as the lever arm changes. We believe that *peak tissue strain rate* would in theory be more robust than *peak velocity* in these conditions and should perform similarly in isometric conditions because the lever arm is fixed.

### Statistical Analysis

To test our first hypothesis, we compared the features of the tissue velocity waveforms before and after the first fatiguing EMS protocol. We collected data for five stimulated muscle twitches before and after the fatiguing protocol. We then normalized the data to their average prefatigue value (i.e., baseline). To assess any initial changes after the fatiguing protocol, we first compared the pooled prefatigue features with the features extracted during the first stimulated muscle twitch using paired *t*-tests. To assess subsequent changes to these waveform features, we then used a repeated measures analysis of variance (rmANOVA) to compare the features extracted over the five muscle twitches following the protocol. We confirmed assumptions of normality using a Shapiro–Wilk test. When we found significant differences, we focused post hoc comparisons on changes from the first muscle twitch using paired *t*-tests with a Holm–Bonferroni correction. Because changes to the waveform features over time might present a subtle pattern of recovery that is not readily captured by a rmANOVA, we also attempted to fit a monotonically increasing trend to the waveform features over time. We chose to use a power regression model ( 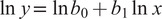 ) to depict a trend because this model exhibited the best fit to our data (e.g., highest *R* ^2^ values) when compared to the other curve fitting models we considered (e.g., linear, logarithmic, and exponential). For the subset of participants who completed the four additional rounds of the fatiguing protocol, we also examined any patterns of recovery after each round by attempting to fit a trend to the resulting waveform features over time.

To assess if and how the waveform features might change over multiple rounds of the fatiguing protocol, we averaged the normalized feature values following each round (five twitches per round) for the subset of participants who completed the four additional rounds. An rmANOVA tested for differences in these pooled features over the five total rounds. We confirmed assumptions of normality using a Shapiro–Wilk test. When we found significant differences, we focused post hoc comparisons on changes from the first round using paired *t*-tests with a Holm–Bonferroni correction.

To test our second hypothesis, we examined the relationship between the torques generated during stimulated muscle twitches and the features of the tissue velocity waveforms. We computed a stepwise multiple linear regression model with the recorded ankle torque as the dependent variable, and the features of the tissue velocity waveforms as covariates. Starting with a constant model, the waveform features were added (*p* < .05) and removed (*p* > .10). Stepwise linear regression iteratively updates a regression model to combine and remove variables systematically to eliminate highly correlated variables and statistically insignificant variables to ensure the data only contains variables with only useful, unique information. To account for any between-subjects variability in the data, we did not normalize the extracted waveform feature values and chose to calculate a separate stepwise regression for each participant. For the participants who completed the four additional rounds of the fatiguing EMS protocol, we included features values from muscle twitches over the five total rounds in their regression analysis.

We performed the statistical analyses using JASP Version 0.16 (JASP Team, Amsterdam, NL) and SPSS Version 27.0 (IBM Corp., Armonk, NY), and defined significance a priori as *p* < .05.

## Results

We recruited 13 participants (9 male, 4 female), and data from nine of these adults included four additional rounds of the fatiguing EMS protocol. We collected data from a total of 479 muscle twitches.

**Figure 4.**
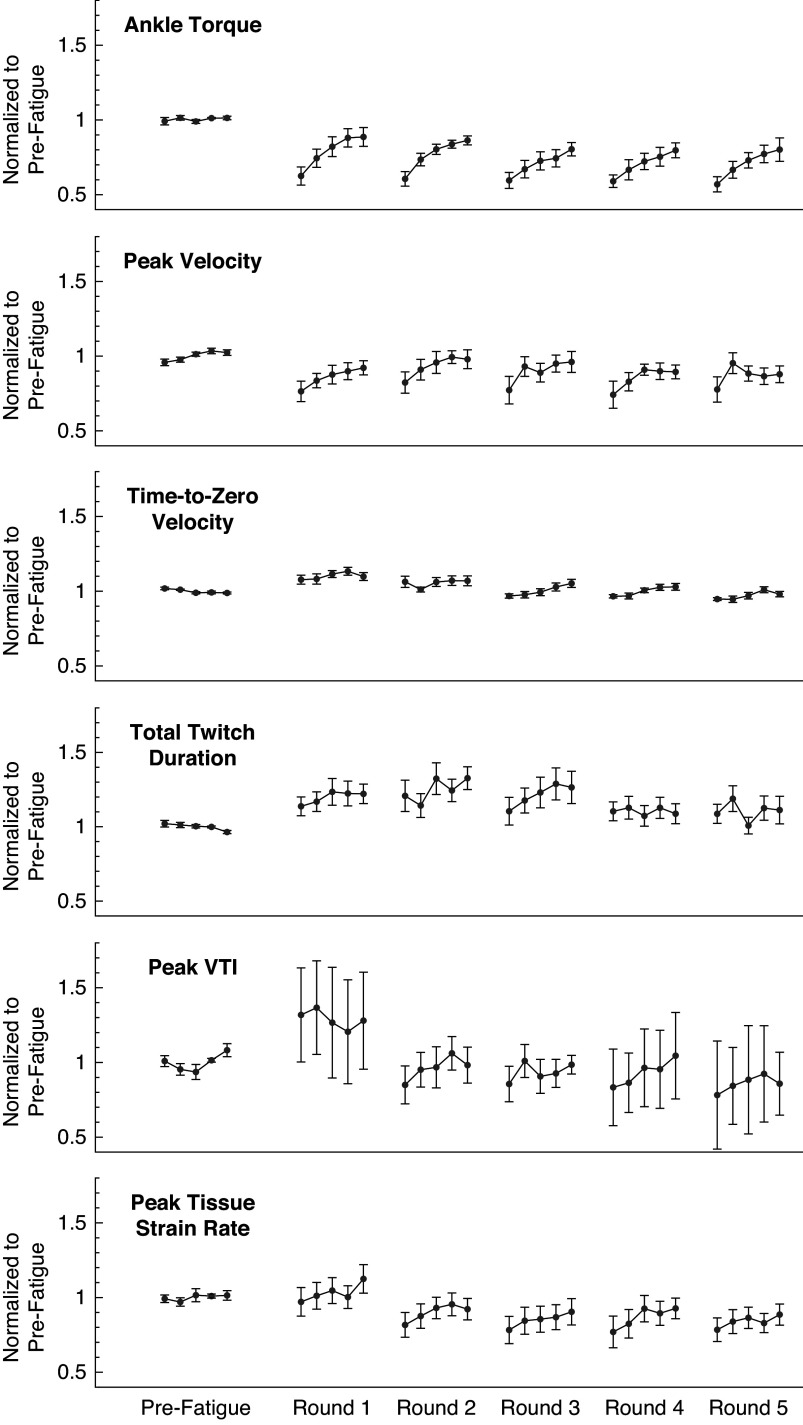
The ankle twitch torque and extracted features of the average muscle tissue velocity waveforms after repeated rounds of the fatiguing stimulation protocol, all normalized to their average pre-fatigue value. Data plotted as mean ± standard error. Data for 13 subjects are included in the plotted values for pre-fatigue and round 1; data for 9 subjects are included in the plotted values for rounds 2 through 5. Note that round 1 refers to the initial application of the fatiguing stimulation protocol, and not the first repetition of the fatiguing stimulation protocol. VTI: Velocity-Time Integral.

We confirmed the onset of fatigue following the first application of the EMS protocol (*N* = 13 participants), in which the normalized ankle torque was significantly reduced during the first stimulated muscle twitch (−38%, *p* < .001, Cohen’s *d* = 2.367) and then recovered over subsequent muscle twitches (Figure 4). The normalized ankle torque significantly changed over the five muscle twitches (*F* = 28.845, *p* < .001, 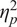 = 0.828). Normalized ankle torque significantly increased from the first muscle twitch during the second (+19%, *p* = .002, *d* = 1.561), third (+31%, *p* < .001, *d* = 2.575), fourth (+41%, *p* < .001, *d* = 3.354), and fifth (+42%, *p* < .001, *d* = 3.434) muscle twitches. Throughout the five muscle twitches during the recovery period, the normalized ankle torque exhibited a monotonically increasing trend that fit a power regression model (*b*_0_ = 0.614, *b*_1_ = 0.236; *F* = 14.504, *R* ^2^ = 0.305, *p* = .001).

We found evidence that normalized features of the tissue velocity waveforms also changed following the fatiguing EMS protocol for the 13 participants (Figure 4). The average feature values generally appeared to follow a similar pattern to the changes in ankle torque, in which the feature values gradually increased from the first stimulated muscle twitch during subsequent muscle twitches. The normalized *peak velocity* was significantly reduced from baseline during the first stimulated muscle twitch (−24%, *p* = .022, *d* = 1.156), and the rmANOVA revealed significant changes over the five muscle twitches (*F* = 4.787, *p* = .007, 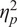 = 0.489). The normalized *peak velocity* significantly increased from the first muscle twitch to the fourth (+23%, *p* = .022, *d* = 1.435) and fifth (+23%, *p* = .022, *d* = 1.413) muscle twitches, but the values for the second (*p* = .843) and third (*p* = .060) muscles twitches were not significantly different. Similar to the normalized ankle torque, the normalized *peak velocity* exhibited a monotonically increasing trend that fits a power regression model. (*b*_0_ = 0.741, *b*_1_ = 0.132; *F* = 4.328, *R* ^2^ = 0.119, *p* = .046). The normalized *time-to-zero velocity* was significantly increased from baseline during the first stimulated muscle twitch (+8%, *p* = .050, *d* = 0.671), but the rmANOVA did not reveal any significant changes over the five muscle twitches (*F* = 2.022, *p* = .115), nor did the data exhibit a significant monotonically increasing trend (*F* = 1.128, *R* ^2^ = 0.022, *p* = .293). The normalized *total twitch duration* was not significantly different than baseline during the first stimulated muscle twitch (+14%, *p* = .064), the rmANOVA did not reveal any significant changes over the five muscle twitches (*F* = 1.136, *p* = .355), and the data did not exhibit a significant monotonically increasing trend (*F* = 1.109, *R* ^2^ = 0.019, *p* = .297). The normalized *peak VTI* was also not significantly different than baseline during the first stimulated muscle twitch (+32%, *p* = .202), the rmANOVA did not reveal any significant changes over the five muscle twitches (*F* = 0.061, *p* = .993), and the data did not exhibit a significant monotonically increasing trend (*F* = 0.211, *R* ^2^ = 0.004, *p* = .648). The normalized *peak tissue strain rate* was not significantly different than baseline during the first stimulated muscle twitch (−3%, *p* = .734), but the rmANOVA did reveal significant changes over the five muscle twitches (*F* = 3.954, *p* = .009, 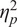 = 0.305). The normalized *peak tissue strain rate* significantly increased from the first muscle twitch to the fifth muscle twitch (+14%, *p* = .011, *d* = 1.118), but the values for the second (*p* = .999), third (*p* = .316), and fourth (*p* = .257) were not significantly different. However, the data for the *peak tissue strain rate* did not exhibit a significant monotonically increasing trend (*F* = 1.467, *R* ^2^ = 0.026, *p* = .231).

For the subset of participants who completed five total rounds of the fatiguing EMS protocol (*N* = 9 subjects), the average waveform features exhibited a similar pattern of recovery after subsequent rounds (Figure 4). The normalized ankle torque during the recovery periods following each round all exhibited a monotonically increasing trend that fit a power regression model (*R* ^2^ = 0.216–0.507, *p* < .010). The normalized *peak velocity* also exhibited this monotonically increasing trend during the recovery periods after the first, third, and fourth round (*R* ^2^ = 0.118–0.143, *p* < .046), but not the second or fifth round (*R* ^2^ = 0.040–0.087, *p* = .068–.266). The normalized *time-to-zero velocity* only exhibited a monotonically increasing trend that fit a power regression model during the recovery periods following the third and fourth rounds (*R* ^2^ = 0.144–0.172, *p* < .019), but not after the first, second, or fifth rounds (*R* ^2^ = 0.007–0.092, *p* = .085–.600). During the recovery periods after all five rounds, the data for the normalized *total twitch duration* (*R* ^2^ = 0.001–0.052, *p* = .119–.925), *peak VTI* (*R* ^2^ = 0.004–0.089, *p* = .069–.648), and *peak tissue strain rate* (*R* ^2^ = 0.007–0.092, *p* = .085–.600) did not exhibit any significant monotonically increasing trends.

We did not observe strong evidence that waveform features would substantially change after multiple rounds of the fatiguing protocol (Figure 4). For example, the rmANOVA did not detect significant changes in the average normalized ankle torque over the five rounds (*F* = 1.506, *p* = .238, 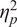 = 0.231). However, an rmANOVA did reveal that the average normalized *time-to-zero velocity* significantly changed across the five rounds (*F* = 17.461, *p* < .001, 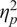 = 0.744). Post hoc comparisons found that the average values from the second (*p* = .012, *d* = 1.310), third (*p* < .001, *d* = 2.080), fourth (*p* < .001, *d* = 2.402), and fifth rounds (*p* < .001, *d* = 2.846) were all significantly lower than the first round. Similarly, an rmANOVA revealed that the average normalized *peak VTI* significantly changed across the five rounds (*F* = 3.065, *p* = .036, 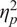 = 0.338), but post hoc comparison only found that the average values from the fifth round were significantly lower than the first round (*p* = .039, *d* = 1.205). The rmANOVA did not detect any significant changes across the five rounds for the average normalized *peak velocity* (*F* = 0.296, *p* = .877, 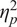 = 0.056), the average normalized *total twitch duration* (*F* = 1.202, *p* = .332, 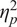 = 0.147), or the average normalized *peak tissue strain rate* (*F* = 1.363, *p* = .272, 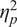 = 0.163).

The stepwise linear regression analysis revealed that features of the velocity tissue waveforms could significantly predict the recorded ankle twitch torque for each individual subject (Table 1). The fit of the regression models varied between subjects (*N* = 9 participants), ranging from *R* ^2^ = 0.255 to *R* ^2^ = 0.849 with a mean of *R* ^2^ = 0.635. We observed that the significant waveform features identified by the stepwise regression were not consistent across the nine participants. The *peak tissue strain rate* emerged as a significant predictor in five of the participant models, the *time-to-zero velocity* emerged in four of the participant models, the *peak VTI* and *total twitch duration* emerged in three participant models, and the *peak velocity* emerged in two participant models.

**Table 1.** Stepwise linear regression to predict ankle torque

	Subject 1 (*R* ^2^ = 0.752)			Subject 2 (*R* ^2^ = 0.849)			Subject 3 (*R* ^2^ = 0.784)		
Model terms	Estimate	Std. error	*p*	Estimate	Std. error	*p*	Estimate	Std. error	*p*
Intercept	2.807	2.169	.212	13.331	1.171	<.001	−6.400	3.119	.052
Peak velocity									
Time-to-zero velocity	61.29	8.307	<.001						
Total twitch duration							6.774	3.205	.046
Peak VTI				52.114	4.159	<.001			
Peak tissue strain rate							10.32	1.173	<.001

	Subject 4 (*R* ^2^ = 0.577)			Subject 5 (*R* ^2^ = 0.477)			Subject 6 (*R* ^2^ = 0.749)		
	Estimate	Std. error	*p*	Estimate	Std. error	*p*	Estimate	Std. error	*p*
Intercept	0.857	0.299	.008	−35.046	10.616	.002	−7.097	1.154	<.001
Peak velocity				1.351	0.312	<.001			
Time-to-zero velocity				356.045	63.822	<.001	76.875	9.506	<.001
Total twitch duration				−13.134	3.739	.001			
Peak VTI	1.912	0.315	<.001						
Peak tissue strain rate				−8.026	3.427	.024	2.596	0.266	<.001

	Subject 7 (*R* ^2^ = 0.666)			Subject 8 (*R* ^2^ = 0.613)			Subject 9 (*R* ^2^ = 0.255)		
	Estimate	Std. error	*p*	Estimate	Std. error	*p*	Estimate	Std. error	*p*
Intercept	6.193	1.192	<.001	1.232	1.897	.519	5.08	1.454	<.001
Peak velocity							3.654	0.828	<.001
Time-to-zero velocity				30.514	11.823	.013			
Total twitch duration	6.168	0.809	<.001						
Peak VTI				−10.621	2.949	<.001			
Peak tissue strain rate	2.805	0.618	<.001	10.944	1.168	<.001			

*Note.* Although we found that features of the velocity tissue waveforms could significantly predict the recorded ankle torque, the significant waveform features identified by stepwise regression were not consistent across subjects.

Abbreviation: VTI, velocity-time integral.

## Discussion

In this study, we examined whether TDI could assess changes in stimulated isometric muscle twitches that are related to muscle fatigue following EMS. We extracted features from the average medial gastrocnemius tissue velocities and found that the *peak velocity* and *time-to-zero velocity* during the first stimulated muscle twitch after a fatiguing EMS protocol had changed significantly from their prefatigue values. We also observed that the *peak velocity*, *time-to-zero velocity*, and *peak tissue strain rate* for five stimulated muscle twitches after the protocol followed the pattern of fatigue recovery exhibited by the isometric ankle torque. We also found that features of the average tissue velocity waveforms could significantly predict the ankle torque for each participant using stepwise linear regression (Table 1). We interpret these results to suggest that TDI can detect changes in muscle tissue during isometric muscle twitch that are related to muscle fatigue, fatigue recovery, and the generated joint torque. Thus, Doppler ultrasound approaches may have utility for monitoring changes to muscle fatigue during applications of EMS and could be considered in the design of future wearable ultrasound feedback devices.

### Tissue Velocity Waveform Features Change after Fatiguing Protocol

In support of our first hypothesis, we found that features of the average tissue velocity waveforms during stimulated muscle twitches had significantly changed after the fatiguing EMS protocol. We used TDI ultrasound to measure features of the muscle tissue velocities, which can indicate muscle activation and release. We anticipated that velocity features would change after fatiguing the muscle because prior studies have found that changes in muscle deformation detected via ultrasound are correlated to isometric output torques (Hallock et al., 2018, 2020). This is understandable due to the varying number of fibers being activated, the nonlinear elastic stretching properties of the tendons in series with the contractile muscle elements (Ito et al., 1998), and the interaction of complex muscle geometries (Lieber and Friden, 2000; Azizi et al., 2008; Azizi and Deslauriers, 2014). However, we only observed that the normalized *peak velocity* and normalized *time-to-zero velocity* exhibited any significant change from their prefatigue value. It may have been that a single round of our fatiguing EMS protocol was not sufficient to evoke detectable changes in the other waveform features, but they could be detected after experiencing an increased level of muscle fatigue. This may explain why subsequent rounds of the protocol exhibited waveform feature values drifting farther away from their prefatigue values (e.g., the *peak tissue strain rate*). We also observed that the *peak velocity*, *time-to-zero velocity*, and the *peak tissue strain rate* over the five stimulated muscle twitches exhibited a pattern of fatigue recovery similar to the ankle torque. Although not significant, we noticed that the other waveform features exhibited a generally similar pattern of gradual increase following a round of stimulation (Figure 4). Again, this pattern of fatigue recovery might be more prominent for the other waveform features after the muscle reaches a sufficient level of fatigue. For example, a pattern of fatigue recovery in the normalized *peak VTI* might not be fully revealed until after four rounds of the fatiguing EMS protocol. Considering that these velocity waveform changes manifested across subjects and throughout repeated rounds of the EMS protocol, we interpret our findings as compelling evidence that TDI has potential to quantify localized changes in muscle fatigue and recovery during EMS applications.

We observed that the average *time-to-zero velocity* and *peak VTI* significantly decreased over five rounds of the fatiguing EMS protocol, but we did not detect a similar decrease in the average isometric ankle torque. We did expect subsequent applications of the fatiguing EMS protocol to elicit a gradual increase in overall muscle fatigue, but this was not reflected in the measured ankle torque. This suggests that TDI might have the sensitivity to detect quantifiable changes in muscle tissue related to localized muscle fatigue that are not as easily detected using a biomechanical assessment of joint torque, which provides only a gross measure of neuromuscular system output and contributes little insight into fatigue within an individual muscle (Vøllestad, 1997). Although the average *peak tissue strain rate* appeared to decrease over five rounds of the protocol, this overall decrease was not statistically significant. This might be due to fiber heterogeneity or boundary conditions affecting the approximated strain (Witte et al., 2006). We also observed an increased variability in the normalized velocity waveform features compared to their prefatigued values, particularly for the *peak VTI* and *total twitch duration* (Figure 4). This might be due to individual subject differences in response to the EMS protocol. For example, some participants may have been more susceptible to muscle potentiation, in which a muscle becomes more sensitive to stimulation after a brief, intense activation (Barry et al., 1992; Sale, 2002). As potentiation and fatigue are both influenced by prior muscle activity but have opposing effects on muscle force production, it can be difficult to quantify either process independently and are thus thought to coexist after initiation of contractile activity (Rankin et al., 1988; Rassier and MacIntosh, 2000).

### Tissue Velocity Waveform Features Predict Twitch Torque during Fatigue Recovery

In support of our second hypothesis, we found that features of the average muscle tissue velocity waveforms during twitch could predict changes in the isometric ankle torque. We were surprised to find that the tissue velocity waveform features identified as significant predictors in the regression were not consistent between subjects. We had anticipated that the *peak tissue strain rate* and the *peak VTI* would emerge as consistent predictors, because they result from net cross-sectional expansion (*peak tissue strain rate* being an indicator of the rate of change of muscle thickness robust to any muscle shifts due to lever arm movement and the *peak VTI* being dependent on the total change in muscle thickness which would be robust to the synchronicity of muscle fiber contractions). The observed between-subjects variability might have been due to anatomical and physiological variations such as in muscle fiber composition, activity level, resting foot progression angle, muscle thickness, length, stiffness, or motor points. We cannot exclude the possibility that our observed relationship between tissue velocities and ankle torque are dependent on the specific orientation and location of the transducer probe. The gastrocnemius is a pennate muscle and thus the tissue location we were measuring was inherently anisotropic. Complicating this further, the pennation angle of the muscle fibers changes during contraction (although it is possible that features related to the time-duration of muscle contraction may be more robust to variations in probe placement). Considering that muscle fiber length may vary with fatigue (Muanjai et al., 2020), we are encouraged that even our relatively simple measures of tissue velocities were sensitive to the changes after EMS, and future studies should consider methods to overcome this between-subjects variability. The strength of this technique is that subject specific combinations of tissue velocity waveform features can still be used to accurately predict muscle twitch torque during fatigue recovery, despite the varying biological factors or experimental conditions such as probe placement. This is important because twitch torque has been found to mirror tetanic force fatigue levels during short durations (<5 min) of fatigue recovery (Baker et al., 1993; Shields et al., 1997).

### Practical Considerations and Potential Applications

The primary motivating factor for investigating ultrasound in EMS applications is that EMS is generally incompatible with the current gold standard for studying voluntary muscle activation, sEMG. This, however, does not limit ultrasound to EMS applications only. In fact, velocity and anatomical information provided by ultrasound is independent of, and complementary to, the electrophysiology information provided by sEMG, which means ultrasound could be used either with or in place of sEMG. Unlike sEMG, ultrasound measurements are depth-resolved, and therefore the specific muscle and its depth are identifiable. A phased array ultrasound beam can be steered, so the source of movement along different directions or depth within the imaging field can be localized. A limitation is that measurements are performed within the probe’s field of view (generally a plane, or a single scan line), which can make it difficult when stimulating muscle groups that are spread farther apart than the field of view, and could possibly require multiple probes. Conversely, the data from an sEMG electrode are not depth-resolved, which means they cannot differentiate signals from multiple overlying muscles, making it difficult to attribute components to their respective source(s). This is particularly a concern in EMS applications because the large stimulus artifacts produced by stimulation can severely corrupt the comparatively small signals generated by the muscle. Because ultrasound provides detailed physiological information without the same susceptibility to EMS artifacts, we believed that it would be an ideal candidate for EMS applications, such as hybrid EMS exoskeletons (Sheng et al., 2019; Zhang et al., 2022a,b).

Our results support our overarching hypothesis that Doppler ultrasound imaging is sensitive to the mechanical signs of muscle fatigue during EMS. More specifically, our results suggest that TDI can detect changes in muscle tissue that are related to muscle fatigue, fatigue recovery, and the generated joint torque. The present study examined a single muscle undergoing isometric contraction due to EMS. Applications of EMS typically include limb movement involving multiple stimulated muscles, and future studies should consider the effect of these potential confounding factors on the recorded muscle tissue velocities. Future studies should also investigate the feasibility of using TDI to monitor changes in muscle fatigue during a fatiguing EMS protocol, instead of limiting the analysis to observed changes during recovery periods following an EMS protocol.

The potential for TDI to monitor muscle fatigue during EMS brings about unique opportunities in biomechanics, rehabilitation, and sports performance. EMS parameters such as timing, duration, and intensity are commonly controlled without any feedback from the targeted muscle (Shields et al., 2006; Freeman et al., 2016; Hallock et al., 2018; Phongamwong et al., 2019), though finding real time feedback to close the loop for human–robot interaction is an active area of research (Zhou et al., 2018; Zhang et al., 2022b). By integrating muscle fatigue as feedback, EMS protocols can be better refined to optimize the desired stimulation outcomes. This might be particularly useful for stimulating muscles effectively over a prolonged period, such as when using a powered exoskeleton coupled with functional EMS (Chang et al., 2022). By gauging the level of fatigue recovery between bouts of EMS, stimulation could be limited to periods when the muscle has recovered enough to generate sufficient torque. Measures of local muscle fatigue might also inform modifications to stimulation parameters (i.e., timing and waveforms), which may increase their effectiveness at reducing EMS-induced fatigue (Binder-Macleod and Kesar, 2005; Graham et al., 2006; Shimada et al., 2006).

Desynchronizing stimulation across multiple sites can be used to mitigate EMS-induced muscle fatigue (Nguyen et al., 2011; Bergquist et al., 2017; Laubacher et al., 2017); thus there is potential for TDI to inform stimulation strategies strategically applied to a set of muscles such that fatigued muscles have sufficient time to recover. There is also potential for TDI to optimize the application of EMS based on the spatial distribution of activated muscle fibers within a muscle. Electrode placement restricts which fibers are activated during EMS, and thus poor electrode placement can lead to accelerated fatigue in certain fibers while leaving other fibers underutilized (Gobbo et al., 2014). Incorporating TDI to characterize EMS/muscle performance might help identify which muscle fibers are activated by certain electrodes to avoid redundant or underutilized placement that would over stimulate, over activate, and prematurely fatigue these fibers. Additionally, by using multiple stimulation sites across a muscle, stimulation protocols could be designed to minimize the demand on the local muscle fibers under each electrode while maintaining a net force from the muscle. Spatially distributed stimulation protocols might be also used to intermittently excite subsets of fibers, which could mimic the natural rest and recruitment patterns of muscle fibers.

We envision future wearable feedback devices can use ultrasound to monitor EMS-induced muscle fatigue in real time. Wearable ultrasound is still an emerging technology (Sikdar et al., 2014; AlMohimeed and Ono, 2020), and we are optimistic that other ultrasound approaches besides TDI may have utility to quantify muscle fatigue (Zhang et al., 2022b). Although TDI provides robust and detailed velocity information, current implementations of TDI typically have high power requirements, impractically large physical forms, extensive computational and memory demands, and are thus generally unsuited for wearable applications. Nonetheless, this study of TDI to quantify muscle fatigue provides a proof of concept that can inform the design of more accessible ultrasound approaches, like continuous wave Doppler imaging (Majdi et al., 2020), which may better serve as the foundation for a real-time, wearable ultrasound system to monitor muscle fatigue. Continuous wave systems can provide velocity estimates from a predefined focal depth. Further, multiple transducers with distinct center frequencies can be used in an array with multiple focal depths to gather depth resolved velocity information, all with significantly reduced memory requirements, power consumption, and sample rates. We are particularly encouraged by the development of frequency-modulated continuous wave (FMCW) ultrasound techniques based on FMCW Doppler radar that might provide low-power, depth-resolved velocity waveforms using a single transducer (Kunita et al., 2010; Tarbox et al., 2017).

### Study Limitations and Future Directions

The study has some limitations to consider. First, the stimulated muscle twitches were assessed during a 2-min rest period at an interval of 20 s. While the data collected were sufficient to demonstrate the potential of assessing fatigue after EMS, further research is needed to assess how the TDI velocity features may change within shorter time windows (i.e., <20 s) or during longer recovery periods (i.e., >2 min). Second, the biomechanical measures of ankle torque in this study represent a measure of the net torque generated at the ankle joint. We stimulated the two heads of gastrocnemius to generate this torque, but only collected Doppler ultrasound images for the medial gastrocnemius. Further work is needed to decompose the individual muscle contributions to the net ankle torque when considering localized measures of muscle fatigue. Third, we did not analyze the effect of subject positioning (prone vs. seated). However, our analysis methods did help to mitigate a potential effect of positioning. Whenever we compared values across subjects, we used normalized values so that any observed changes were relative to their prefatigue values. During the regression analyses, we did not pool the subject’s data together and instead calculated an individual regression for each subject, which kept any positioning effect consistent for that subject. Fourth, because muscle fatigue is a complex, multifactorial process involving various central and peripheral mechanisms (Dugan and Frontera, 2000), there may be additional underlying factors contributing to our observed changes in ankle torque (Rassier and MacIntosh, 2000).

Further research should determine if the observed changes in ultrasound imaging is consistent across a variety of stimulation patterns and protocols. Another limitation is that the TDI method only measures the axial component of tissue velocity (center dotted line in Figure 1c) while the muscle shortening occurs along the fiber direction. The true shortening velocity is related to the estimated axial velocity by the cosine of the angle between the ultrasound beam and the fiber direction. The pennation angle observed in ultrasound B-mode images of the medial gastrocnemius muscle at rest provides an initial estimate of the fiber direction, however, the pennation angle changes during the contraction. While the rate of change of the pennation angle may be small during an isometric contraction (Héroux et al., 2016), the relationship between the estimated axial TDI velocity and the true shortening velocity is nevertheless complex. Thus, the *peak velocity* feature needs to be interpreted with caution since it is not directly proportional to peak shortening velocity. We also note that in our study, we did not find that the observed relationship between axial velocity and torque was dependent on the initial pennation angle at rest.

## Conclusion

In conclusion, we found that tissue Doppler ultrasound imaging during stimulated isometric muscle twitches after EMS can detect changes in muscle fatigue, fatigue recovery, and the generated joint torque. Thus, TDI may have utility to monitor EMS-induced muscle fatigue. These findings support the potential of using TDI approaches with wearable ultrasound to develop a real-time, wearable muscle fatigue monitor compatible with EMS.

## Data Availability

The data that support the findings of this study are available from the corresponding author, S.S., upon reasonable request.

## Notations

EMS: **electrical muscle stimulation**
FMCW: **frequency-modulated continuous wave**
I_20_: **stimulation current required to generate 20% of the maximum voluntary isometric contraction**
MVIC: **maximum voluntary isometric contraction**
rmANOVA: **repeated measures analysis of variance**
sEMG: **surface electromyography**
TDI: **tissue Doppler imaging**
VTI: **velocity-time integral**
